# The empty roof sign: A new indirect magnetic resonance imaging sign of posterior cruciate ligament injury

**DOI:** 10.1002/jeo2.70805

**Published:** 2026-06-15

**Authors:** Amanda Magosch, Natalie Mengis, Maximiliano Ibañez, Christian Nührenbörger, Caroline Mouton, Felix Hoffmann, Caroline Chabot, Romain Seil

**Affiliations:** ^1^ Department of Sports Medicine and Prevention/Department of Orthopedic Surgery Centre Hospitalier de Luxembourg – Clinique d'Eich Luxembourg Luxembourg; ^2^ Luxembourg Institute of Research in Orthopaedics, Sports Medicine and Science (LIROMS) Luxembourg Luxembourg; ^3^ University Department of Orthopedic Surgery and Traumatology Kantonsspital Baselland Bruderholz Switzerland; ^4^ Department of Clinical Research, Research Group Michael T. Hirschmann, Regenerative Medicine & Biomechanics University of Basel Basel Switzerland; ^5^ Catalan Institute of Traumatology and Sports Medicine, Institut Català de Traumatologia I Medicina de L'Esport (ICATME), Hospital Universitari Quiron‐Dexeus Barcelona Spain; ^6^ Department of Radiology Cliniques Universitaires Saint‐Luc, Université Catholique de Louvain Brussels Belgium; ^7^ Human Motion, Orthopaedics, Sports Medicine and Digital Methods (HOSD) Luxembourg Institute of Health (LIH) Luxembourg Luxembourg

**Keywords:** empty roof sign, knee, magnetic resonance imaging, partial injury, posterior cruciate ligament, posteromedial bundle

## Abstract

**Purpose:**

Posterior cruciate ligament (PCL) injuries can pose diagnostic challenges. Magnetic resonance imaging (MRI) is an important diagnostic tool, yet direct visualization of partial or chronic PCL tears is limited, and reliable indirect imaging signs are not well established. The aim of this study was to describe a novel indirect MRI sign of PCL injury and to evaluate its occurrence in a retrospective case series of patients with and without intact PCL.

**Methods:**

The ‘empty roof sign’ (ERS) was defined as an indirect MRI finding on sagittal T2‐weighted fat‐suppressed images, characterized by a fluid‐filled triangular gap between the ACL and the intercondylar notch roof. In a retrospective case series, patients with arthroscopically confirmed partial or complete PCL injuries who underwent surgery were compared to patients with arthroscopically intact PCL who underwent surgery for reasons other than knee ligament reconstruction. Preoperative MRIs were reviewed for the presence of the ERS. Diagnostic performance was assessed using arthroscopy as the reference standard.

**Results:**

The retrospective review of 20 patients with arthroscopically confirmed partial or complete PCL injuries demonstrated a positive ERS on the preoperative MRI in 80% of cases. Notably, all patients with partial PCL injuries exhibited the ERS despite preserved PCL morphology on MRI. In a comparator case series of 22 patients with intact cruciate ligaments, the ERS was present in only 9% of cases. The ERS showed a sensitivity of 80% and specificity of 91%, with a positive predictive value of 89% and a negative predictive value of 83%, yielding an overall accuracy of 86%. The difference between the two case series cohorts was statistically significant (*p* < 0.001).

**Conclusions:**

Findings from this case series suggest that the ERS may serve as a promising marker of PCL insufficiency, particularly in partial PCL injuries where the ligament appears morphologically intact on the conventional MRI.

**Level of Evidence:**

Level IV, retrospective case series.

**Abbreviations**
ACL: anterior cruciate ligament
CI: confidence Interval
ERS: empty roof sign
IQR: interquartile range
MRI: magnetic resonance imaging
PCL: posterior cruciate ligament
PMB: posteromedial bundle

## INTRODUCTION

Posterior cruciate ligament (PCL) injuries predominantly result from high‐energy trauma and are frequently part of multiligamentous injury patterns [14, 19]. Isolated PCL injuries are considerably less common [14, 19, 34]. They typically result from mechanisms including a direct trauma to the anterior aspect of the proximal tibia in a flexed knee position, but may also result from hyperextension, hyperflexion or rotational forces [9, 20, 21, 34, 37]. These less obvious isolated injuries are prone to underdiagnosis and require a high level of clinical suspicion [15, 34].

Clinical examination of PCL injuries can be challenging because symptoms are nonspecific or subtle, and individual physical tests, such as the posterior drawer test at 90° of knee flexion, show variable sensitivity and specificity [17, 20]. Magnetic resonance imaging (MRI) is the gold standard imaging modality for PCL injuries and shows high accuracy for acute tears, but lesion detection remains challenging, especially in partial or chronic cases [26]. On sagittal T2‐weighted fat‐suppressed sequences, acute PCL tears may demonstrate fibre discontinuity, intra‐ligamentous high signal or abnormal ligament contour, with or without avulsion at the tibial or femoral attachment sites [12, 29]. However, the PCL often demonstrates an intermediate or increased intrasubstance signal and focal thickening without frank disruption, particularly in partial tears or chronic injuries, and may even appear continuous despite functional insufficiency [25, 36]. This can limit the reliability of direct signs alone in determining the exact extent of the tear [4]. Because of these limitations, indirect MRI signs have been explored to support the diagnosis of PCL lesions. Secondary findings can include passive posterior tibial translation, especially in the medial compartment [5, 6], protrusion of the anterior medial meniscus in the medial compartment [2], periligamentous or posterior capsular oedema, an increased anteroposterior diameter (≥8 mm) of the PCL [23, 27, 29], and bone marrow contusions in the lateral or patellofemoral compartments [3, 11]. While none of these secondary signs is fully sensitive or specific in isolation, their presence can provide valuable clues to PCL insufficiency, particularly when interpreted alongside clinical assessment [6].

Still, the diagnostic evaluation of PCL injuries remains challenging, particularly in chronic cases and low‐grade or partial tears. While MRI morphologic features are well established for ACL injuries [10], evidence supporting indirect or subtle MRI signs for PCL injury is still limited. Given the biomechanical role of the PCL as the primary restraint to posterior tibial translation [1, 18, 34], assessment of sagittal‐plane alignment may provide valuable indicators of ligament insufficiency.

The aim of this study was to describe a novel indirect MRI sign of PCL injury, termed the ‘empty roof sign’ (ERS), and to evaluate its occurrence in a retrospective case series. It was hypothesized that the ERS would occur significantly more frequently in patients with PCL injury than in a comparator cohort with intact PCL.

## METHODS

### Definition of the ERS

**Figure 1:**
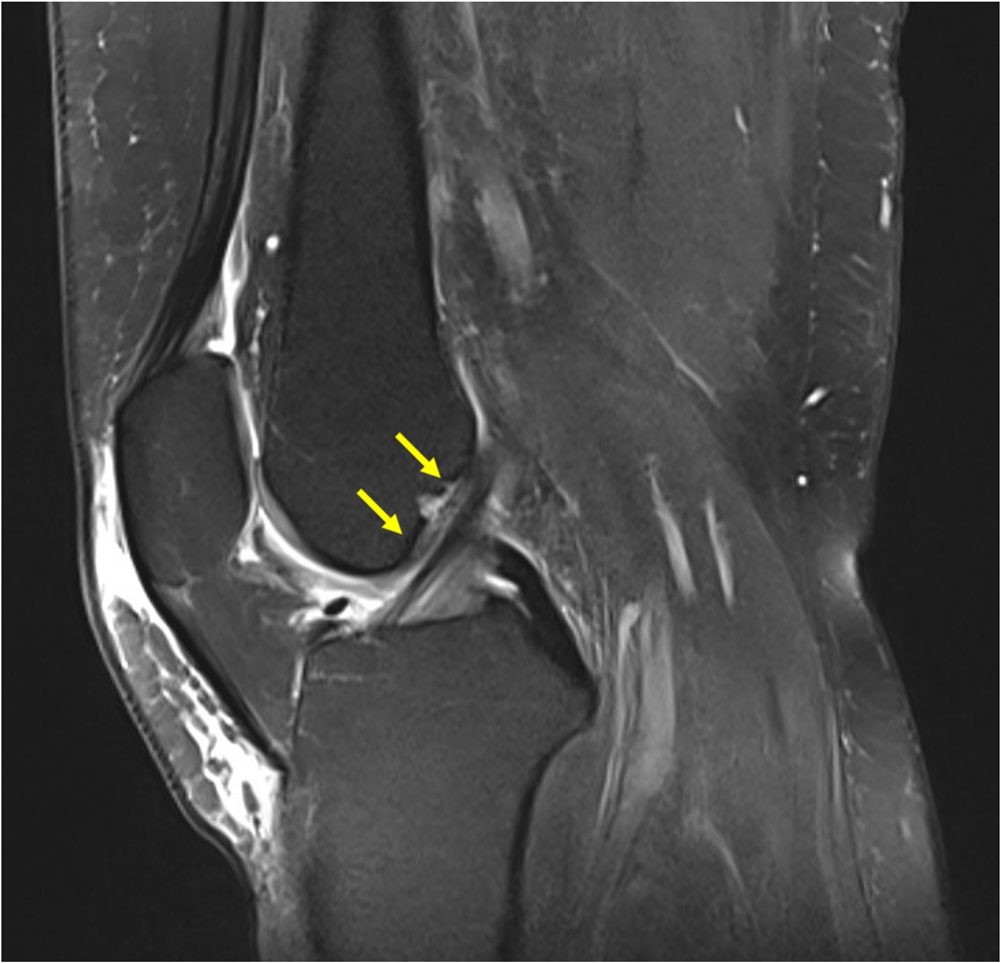
Sagittal fat‐suppressed T2‐weighted MR image of a patient with intact cruciate ligaments. The ACL runs parallel to the ‘Blumensaat line’ and maintains close contact with the roof of the intercondylar notch (→). ACL, anterior cruciate ligament; MR, magnetic resonance.

**Figure 2:**
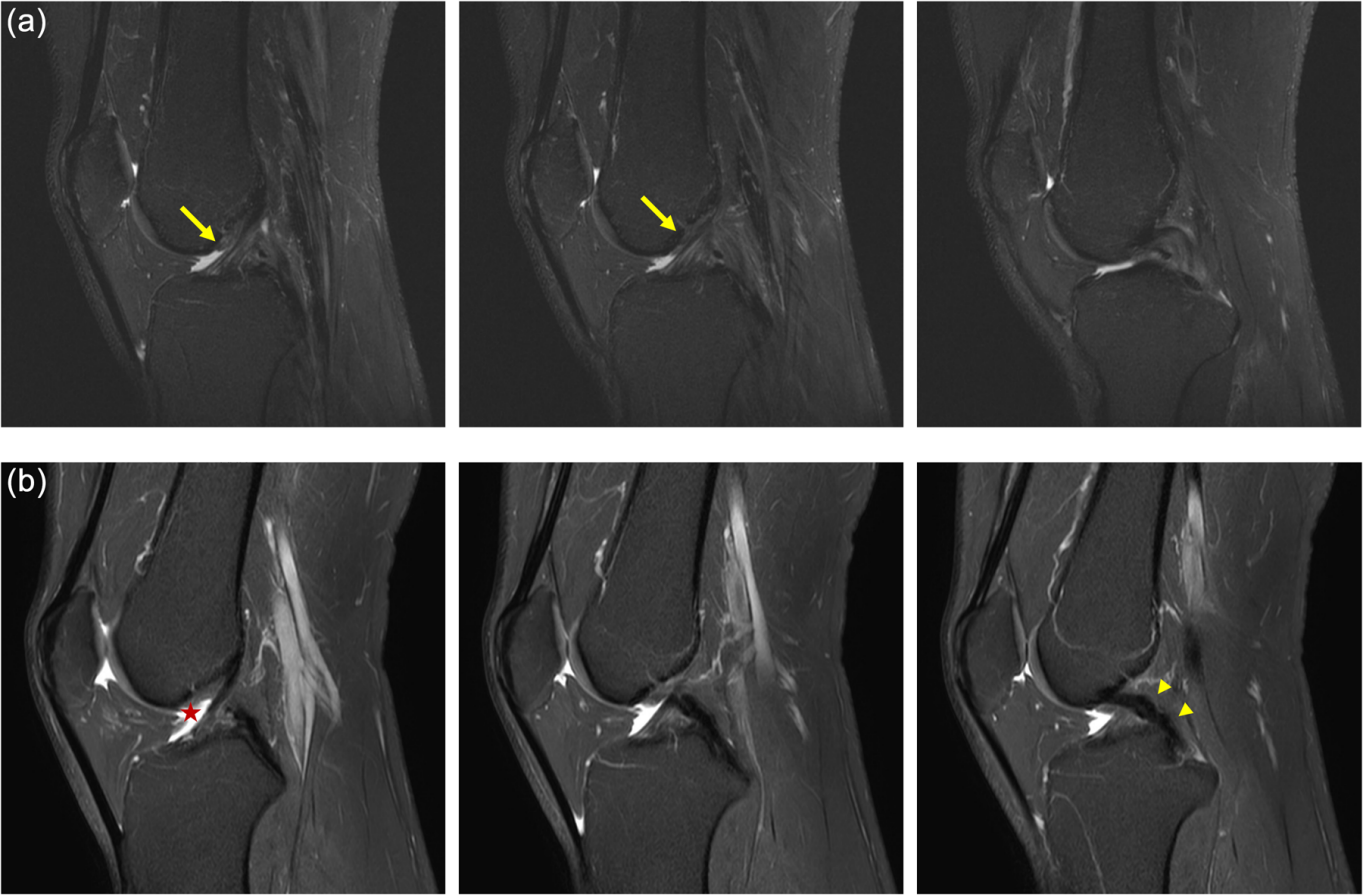
Sagittal fat‐suppressed T2‐weighted MR images of a single patient at two time points. (a) MR images obtained prior to PCL injury: the ACL is in close contact with the roof of the intercondylar notch; the PCL is intact with a normal course. (b) MR images acquired after partial PCL injury: the ‘empty roof sign’ is now positive (★); the PCL appears continuous (►), but compared with the previous examination, a subtle alteration in its course is evident. ACL, anterior cruciate ligament; MR, magnetic resonance; PCL, posterior cruciate ligament.

In a series of patients presenting with clinical signs of PCL injury or insufficiency, the authors identified a frequently observed, previously undescribed imaging finding on routine preoperative MRI, designated the ERS. It is proposed as a novel indirect MRI indicator of primary PCL insufficiency, observed on sagittal T2‐weighted fat‐suppressed sequences of the extended or near‐extended knee. In a knee with intact cruciate ligaments, the ACL appears as a taut, continuous band running obliquely from the posteromedial aspect of the lateral femoral condyle downward and forward to its tibial attachment, with an inclination parallel or slightly more vertical relative to the intercondylar notch roof (*Blumensaat line*) [32]. The ACL closely follows the contour of, and remains in apposition with, the intercondylar notch roof (fossa intercondylaris femoris) (Figures 1 and 2a).

**Figure 3:**
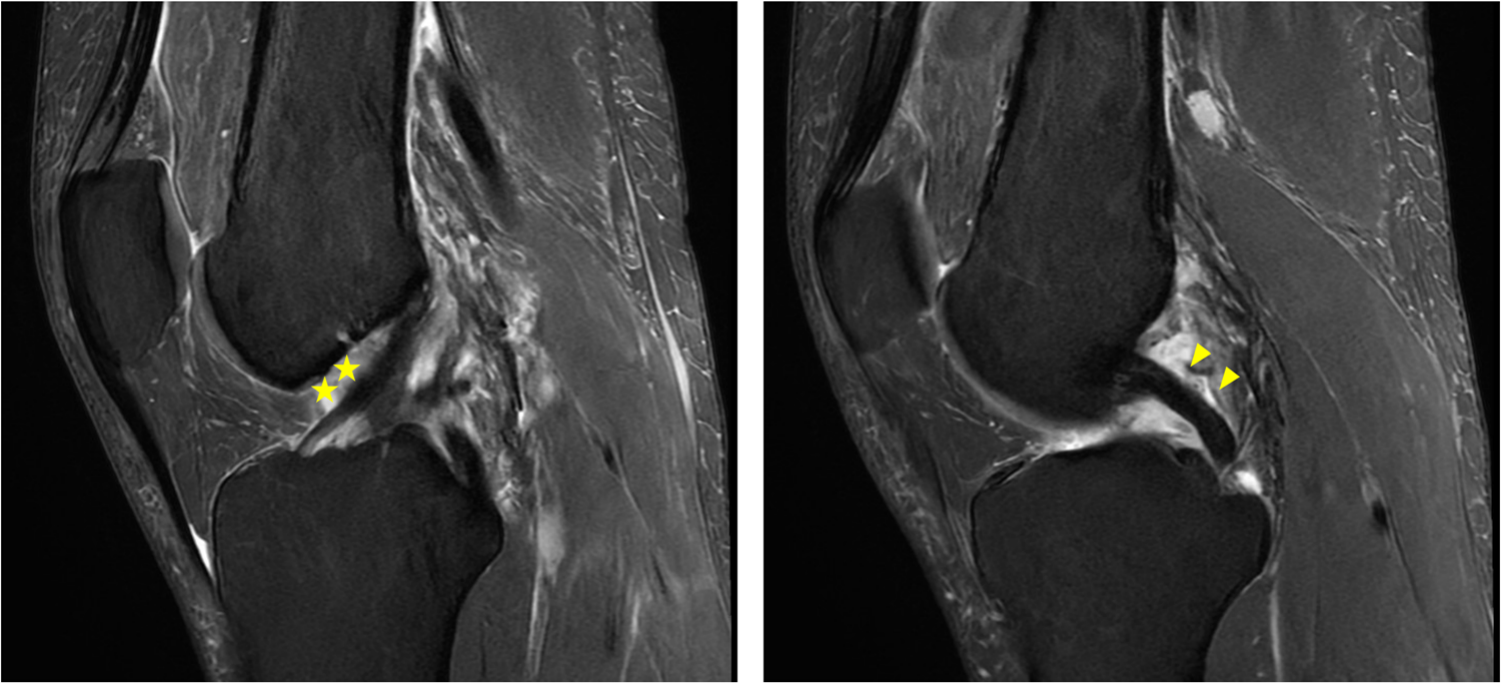
Sagittal fat‐suppressed T2‐weighted MR images of a patient with a partial PCL injury. Depiction of a positive ‘empty roof sign’ (★). The PCL appears continuous (►). ACL, anterior cruciate ligament; MR, magnetic resonance; PCL, posterior cruciate ligament.

**Figure 4:**
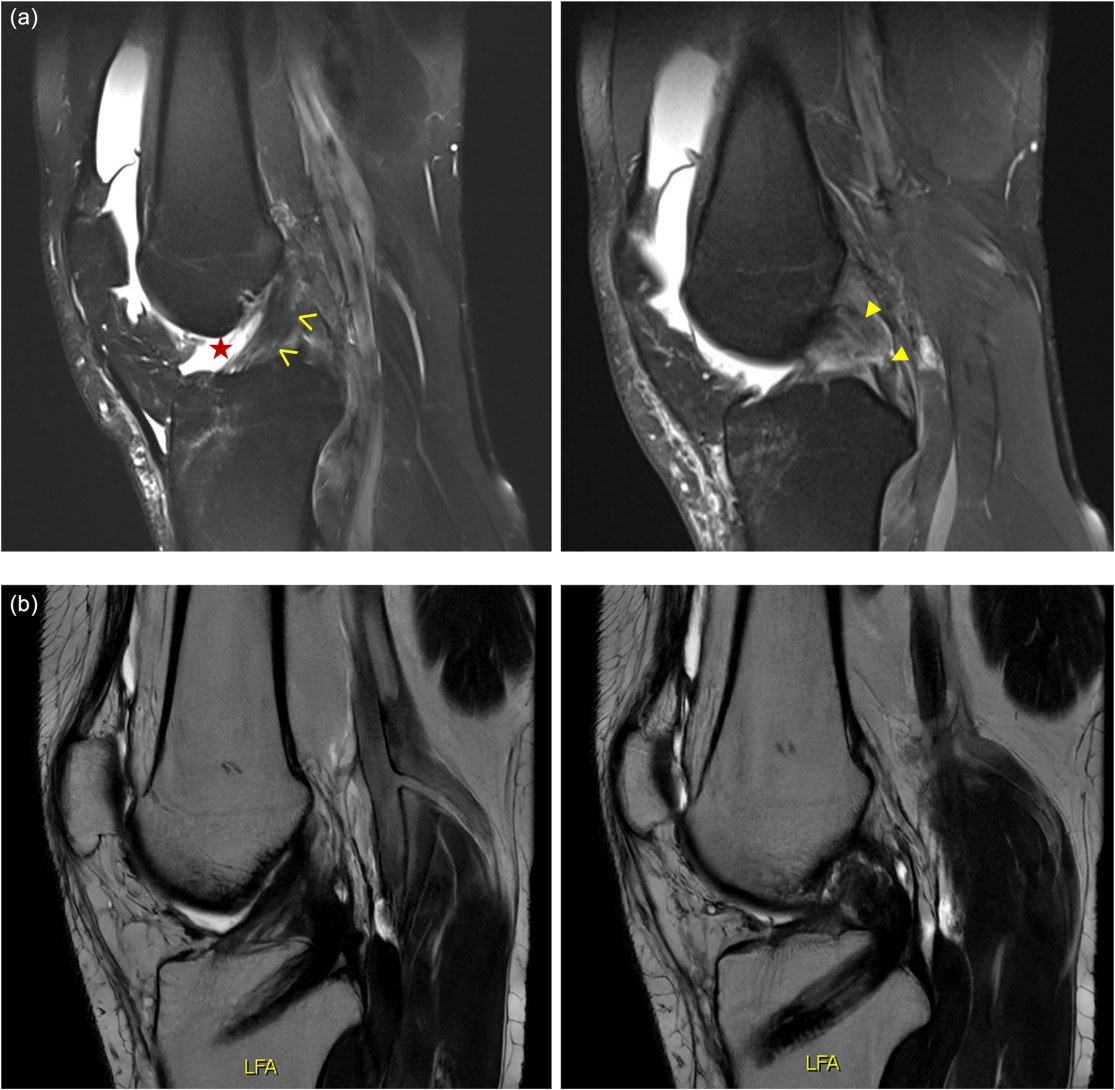
Sagittal MR images of a patient with a PCL injury. (a) Fat‐suppressed T2‐weighted MR images obtained prior to surgery: depiction of a positive ‘empty roof sign’ (★) with a sagging and undulating configuration of the ACL (<); the PCL is fully ruptured (►). (b) T2‐weighted MR images obtained after PCL reconstruction surgery: the ‘empty roof sign’ is negative and the PCL is reconstructed. ACL, anterior cruciate ligament; MR, magnetic resonance; PCL, posterior cruciate ligament.

In a PCL‐deficient knee, the ACL remains structurally continuous on sagittal imaging. However, posterior tibial translation resulting from PCL insufficiency may disrupt the normal close apposition of the ACL to the intercondylar notch roof. On sagittal MRI, this produces a triangular space filled with fluid between the superior surface of the ACL and the intercondylar notch roof, which is referred to as the ‘empty roof’ (Figures 2b and 3). To avoid false‐positive interpretation of the ERS, assessment should not be limited to spatial relationships alone but should also include evaluation of ACL tension and morphology. In cases of a questionable positive ERS, the presence of a sagging, undulating, or lax configuration may provide supportive evidence and assist in diagnostic confirmation (Figure 4a).

The ERS should be assessed on sagittal slices that ideally demonstrate the entire course of the ACL from its femoral to its tibial insertion. Depending on MRI slice thickness, this may be visible on a single slice or across multiple consecutive slices. Table 1 summarizes the authors' constraints for ERS assessment, defining the clinical scenarios in which the sign should not be applied or should be interpreted with caution. Small synovial recesses and joint effusion or hemarthrosis may increase fluid signal within the intercondylar notch, especially after acute trauma. However, these findings do not produce a consistent geometric alteration of the relationship between the ACL and the intercondylar notch roof. In contrast, the ERS represents a reproducible triangular configuration with loss of ACL‐notch roof contact and relative ACL laxity caused by posterior tibial translation in PCL insufficiency.

**Table 1:** Constraints for ‘empty roof sign’ (ERS) assessment.

Constraint	Authors' justification
ACL pathology	The presence of an ACL injury precludes reliable assessment of the ERS, as structural disruption or altered fibre morphology invalidates its interpretation.
Prior ACL reconstruction	Prior ACL reconstruction may alter ligament trajectory and signal characteristics, limiting applicability of the sign.
Inappropriate MRI quality	Thick slices or suboptimal spatial resolution may impair visualization of the ACL relative to the femoral intercondylar notch, increasing the risk of misinterpretation.
Pain‐related guarding (acute injury phase)	Pain‐related muscular guarding in the very acute post‐traumatic phase may reduce posterior tibial translation, potentially leading to a false‐negative ERS.
Significant soft tissue swelling/joint effusion (acute injury phase)	Marked soft tissue swelling or joint effusion in early post‐traumatic examinations may reduce posterior tibial translation, potentially leading to a false‐negative ERS.

Abbreviations: ACL, anterior cruciate ligament; MRI, magnetic resonance imaging.

### Retrospective exploratory clinical case series

To explore the hypothesis that the ERS represents an indirect imaging sign of PCL insufficiency, a retrospective case series was created. The National Ethics Committee for Research in Luxembourg confirmed that this case series did not require formal ethical approval (registration number: 2026‐EA‐13).

Initially, a case series of patients with arthroscopically confirmed partial or complete PCL injury was established. Patients who underwent primary complete or partial PCL reconstruction at our institution between March 2017 and July 2025 were identified through the hospital's surgical database. Patients were excluded if preoperative MRI of the injured knee was not available for review. According to the constraints for ERS assessment (Table 1), patients who underwent ACL reconstruction or had a history of prior ACL reconstruction (*n* = 43) and patients demonstrating extensive soft tissue swelling with pronounced fluid accumulation or joint effusion on MRI (*n* = 3) were excluded.

Subsequently, a comparator case series comprising patients with arthroscopically confirmed intact PCL was established. For this purpose, patients who underwent knee arthroscopy for indications other than ligament reconstruction at our institution between January and July 2025 were identified using the hospital's surgical database. For all patients during diagnostic arthroscopy, an intact PCL was documented. Patients were excluded if preoperative MRI of the injured knee was not available for review. Further, patients with concomitant ligament reconstruction procedures or a history of cruciate ligament reconstruction and patients demonstrating extensive soft tissue swelling with pronounced fluid accumulation or joint effusion on MRI were excluded.

For both case series cohorts, patient characteristics, including sex, age at the time of MRI, the interval between MRI and surgery, and the performed surgical procedure, were extracted in fully anonymized form by a physician with authorised access to the medical records. Categorical variables (sex and surgical procedure) were reported as absolute frequencies and percentages. Continuous variables (age at the time of MRI, interval between MRI and surgery) were reported as medians with interquartile ranges (IQRs), as normality of the data was not assumed.

MRI examinations were either performed at the institution where the surgical procedures were conducted or acquired externally. Consequently, no standardized imaging protocol was applied. The preoperative sagittal T2‐weighted fat‐suppressed MRI scans of all patients in both cohorts were reviewed in consensus by two experienced authors (A.M. and R.S.) to assess the presence of an ERS. Blinding to the clinical diagnosis was not possible, as the PCL status and other intra‐articular pathologies (e.g., meniscal injuries in the comparator cohort) were readily identifiable on image review.

### Diagnostic performance of the ERS

The diagnostic performance of the ERS for the detection of PCL insufficiency was assessed using standard measures of diagnostic accuracy, with arthroscopically confirmed PCL status serving as the reference standard. Sensitivity, specificity, positive predictive value, negative predictive value, and overall diagnostic accuracy were calculated. Exact 95% confidence intervals (CIs) for all proportions were calculated using the Clopper–Pearson method. Comparisons of ERS frequency between the PCL‐deficient and the comparator case series cohorts were performed using Fisher's exact test. All tests were two‐sided, and statistical significance was set at *p* < 0.05. Given the exploratory nature of this retrospective case series, no adjustment for multiple comparisons was applied. Statistical analyses were performed using Python 3.11.4 with SciPy version 1.10.1, Statsmodels version 0.14.0 and Pandas version 1.5.3.

## RESULTS

The case series of patients with arthroscopically confirmed partial or complete PCL injury included 20 patients. Two patients (10%) underwent isolated PCL reconstruction. Three patients (15%) underwent PCL reconstruction combined with medial collateral ligament reconstruction. Nine patients (45%) underwent PCL reconstruction in combination with reconstruction of the posterolateral corner of the knee. One patient (5%) with a tibial avulsion of the PCL underwent transosseous refixation combined with posterolateral corner reconstruction. Five patients (25%) had a rare partial PCL injury, in which isolated reconstruction of the posteromedial bundle (PMB) of the PCL was performed [16, 22]. The comparator case series included 22 patients. Two patients (9%) underwent articular debridement, and 20 patients (91%) underwent meniscal surgery (partial resection or meniscus repair). Table 2 presents the patient characteristics of the two cohorts.

**Table 2:** Patient characteristics of the two case series cohorts.

Patient characteristic	PCL‐injured patients (*n* = 20)	Comparator patients (*n* = 22)
Sex (male/female)	14/6	12/10
Median age at the time of MRI (years)	20 (IQR: 16–38.5)	50.5 (IQR: 43.5–59.75)
Median time from MRI to surgery (days)	88.5 (IQR: 47–177.75)	183.5 (IQR: 88.25–278.5)

Abbreviations: IQR, interquartile range; MRI, magnetic resonance imaging; PCL, posterior cruciate ligament.

A positive ERS was identified on preoperative MRI in 16 cases (80%) with arthroscopically confirmed PCL injury. Notably, all five patients (100%) with partial PCL injury undergoing PMB augmentation displayed a positive ERS, despite otherwise preserved PCL morphology on MRI. Among the four cases with a negative ERS, there were two patients with isolated PCL injuries, one patient with a tibial avulsion of the PCL and a posterolateral corner injury, and one patient with a combined PCL and medial collateral ligament injury. In the comparator case series, a positive ERS was identified on preoperative MRI in two cases (9%).

The ERS demonstrated a sensitivity of 80% (16/20; 95% CI: 56–94) and a specificity of 91% (20/22; 95% CI: 71–99). The positive predictive value was 89% (16/18; 95% CI: 65–99), and the negative predictive value was 83% (20/24; 95% CI: 63–95). Overall diagnostic accuracy was 86% (36/42; 95% CI: 71–95). Fisher's exact test revealed a statistically significant difference in ERS frequency between the two case series cohorts (*p* < 0.001).

## DISCUSSION

The main finding of the present study is the presence of an ERS on sagittal T2‐weighted fat‐suppressed MRI sequences, which may represent a novel indirect sign corresponding to PCL insufficiency. To the author's knowledge, this is the first description of this radiological sign. In the present case series, a positive ERS was detected in 80% of PCL‐injured knees compared with 9% in patients with intact cruciate ligaments. These findings may suggest that the ERS could complement existing MRI diagnostic criteria in patients with PCL insufficiency and potentially address limitations in cases where the PCL appears morphologically intact.

Remarkably, the ERS was positive in all patients with partial PCL injury undergoing surgical augmentation of the PMB, using the technique previously published by Ibañez et al. [16]. These patients presented with inconclusive clinical symptoms and experienced delayed diagnosis despite evaluation by multiple physicians. MRI consistently demonstrated an intact PCL, rendering the diagnosis non‐obvious and ultimately established only following evaluation by the senior author. Such injuries are receiving increasing scientific and clinical attention [28]. A rare clinical symptom constellation for PMB injuries after knee hyperextension trauma—including increased posterior laxity near full extension, which normalizes at 90° of knee flexion, and increased hyperextension compared to the contralateral knee—was recently reported by Mouton et al. [22]. The findings of this case series suggest that the ERS may be particularly useful for detecting functionally relevant PCL insufficiency in these partial PCL injuries, and its integration into clinical decision‐making could enhance diagnostic confidence and detection.

Sagittal tibiofemoral alignment in MRI has already been investigated in ACL injuries, where it provides valuable indirect imaging signs for diagnosis. In ACL deficiency, alignment changes and secondary alterations in ligament configuration, quantified by MRI parameters such as the PCL posterior cortex angle, have proven useful for assessing altered knee biomechanics [7, 24, 31]. In contrast, evidence for such alignment‐based markers in PCL injuries remains limited. However, similar principles may apply to PCL injuries and warrant systematic evaluation.

From an anatomical and biomechanical perspective, the ERS is plausible. The PCL restricts posterior tibial translation, and current evidence supports a codominant role of the two PCL bundles across the entire range of knee motion; neither bundle serves as the sole primary restraint at any particular flexion angle [1, 18, 34]. Some studies suggest that the PMB is taut near extension and contributes to restraint of posterior tibial translation in low flexion and hyperextension, whereas the anterolateral bundle remains relatively slack in full extension and becomes taut beyond approximately 40° of knee flexion, providing restraint at higher flexion angles [8, 13]. Since MRI examinations are typically performed with the knee in extension or near‐extension, a positive ERS may therefore indicate an isolated PMB injury or involvement of the PMB in higher‐grade PCL lesions. Further biomechanical studies are required to validate this assumption.

PCL deficiency is associated with an increased risk of secondary joint damage, particularly involving the meniscus and articular cartilage [33]. Both operative and conservative treatments have been shown to provide satisfactory clinical outcomes when the injury is correctly diagnosed and appropriately managed. Surgical reconstruction may improve mechanical stability and potentially reduce the risk of secondary osteoarthritis in patients with persistent instability, whereas conservative treatment can yield good functional results in low‐grade injuries and selected patient populations [30, 35].

Several limitations should be considered. Given the retrospective and exploratory nature of this study, the reported findings should be interpreted as hypothesis‐generating and require validation in larger, prospective, and blinded cohorts with evaluation of inter‐ and intra‐rater reliability. Hence, the diagnostic performance reported here should be interpreted with caution. The small cohort size results in a wide 95% CI. In this study, MRI assessments were performed with knowledge of the patients' clinical background, including medical history, symptoms, clinical examination and arthroscopic findings, which introduces potential bias. However, this approach reflects real‐world clinical practice, where conclusions are based on an integrated evaluation of clinical and imaging data. The orientation of the MRI examinations and knee flexion angle was not standardized, which may have influenced ERS visibility. This limitation also reflects an issue frequently encountered in clinical practice, where MRI protocols are heterogeneous and clinical suspicion for PCL insufficiency may not be the primary diagnostic consideration, potentially limiting protocol optimization from a radiological perspective. It is conceivable that the ERS may occur at different frequencies in acute versus chronic injuries, but data regarding the interval between injury and MRI or surgery were unavailable in most cases, precluding analysis. In addition, concomitant ligamentous or meniscal injuries in patients with PCL injuries may also influence the presence or appearance of the ERS. Similarly, the comparator case series consisted of patients undergoing arthroscopy for other indications and may not fully represent a healthy population, yet it provides a clinically relevant comparator.

Nevertheless, the ERS has the potential to improve timely and confident diagnosis of PCL injury, particularly in partial or chronic lesions that are often challenging to detect, thereby reducing underdiagnosis of functionally relevant PCL insufficiency. Validation of the ERS in larger, methodologically rigorous studies could support its use as an adjunctive imaging sign with potential implications for clinical decision‐making and patient outcomes.

## CONCLUSIONS

Findings from this case series suggest that the ERS may serve as a promising marker of PCL insufficiency, particularly in partial PCL injuries where the ligament appears morphologically intact on conventional MRI.

## AUTHOR CONTRIBUTIONS

The ‘empty roof sign’ was first noted by Romain Seil. Amanda Magosch, Natalie Mengis and Romain Seil made substantial contributions to the conception, description and study design. Amanda Magosch, Natalie Mengis, Maximiliano Ibañez, Felix Hoffmann and Romain Seil screened and evaluated the MR images. Caroline Chabot contributed valuable insights from a radiological perspective. All authors were involved in drafting or critically revising the manuscript. Each author approved the final version for publication and agrees to be accountable for all aspects of the work, ensuring that questions related to accuracy or integrity are appropriately investigated and resolved.

## FUNDING

The authors have no funding to report.

## CONFLICT OF INTEREST STATEMENT

The authors declare no conflicts of interest.

## ETHICS STATEMENT

The National Ethics Committee for Research in Luxembourg (Comité National d'Ethique de Recherche) confirmed that the publication of this case series does not require an opinion from the ethical committee (registration number: 2026‐EA‐13).

## Data Availability

The data that support the findings of this study are available from the corresponding author upon reasonable request.

## References

[1] Ahmad CS , Cohen ZA , Levine WN , Gardner TR , Ateshian GA , Mow VC . Codominance of the individual posterior cruciate ligament bundles. An analysis of bundle lengths and orientation. Am J Sports Med. 2003;31:221–225. 12642256 10.1177/03635465030310021101

[2] Ahn DY , Park HJ , Kim MS , Kim JN , Hong SW , Kim E , et al. Protruding anterior medial meniscus and posterior tibial translation as secondary signs of complete and partial posterior cruciate ligament tear. Br J Radiol. 2022;95:20210976. 35138916 10.1259/bjr.20210976 PMC10993969

[3] Ali AM , Pillai JK , Gulati V , Gibbons CER , Roberton BJ . Hyperextension injuries of the knee: do patterns of bone bruising predict soft tissue injury? Skeletal Radiol. 2018;47:173–179. 28856482 10.1007/s00256-017-2754-y

[4] Boks SS , Vroegindeweij D , Koes BW , Hunink MGM , Bierma‐Zeinstra SMA . Follow‐up of posttraumatic ligamentous and meniscal knee lesions detected at MR imaging: systematic review. Radiology. 2006;238:863–871. 16452395 10.1148/radiol.2382050063

[5] Degnan AJ , Maldjian C , Adam RJ , Harner CD . Passive posterior tibial subluxation on routine knee MRI as a secondary sign of PCL tear. Radiol Res Pract. 2014;2014:715439. 25587446 10.1155/2014/715439 PMC4283255

[6] DePhillipo NN , Cinque ME , Godin JA , Moatshe G , Chahla J , LaPrade RF . Posterior tibial translation measurements on magnetic resonance imaging improve diagnostic sensitivity for chronic posterior cruciate ligament injuries and graft tears. Am J Sports Med. 2018;46:341–347. 29028358 10.1177/0363546517734201

[7] Di Maria F , D'Ambrosi R , Sconfienza LM , Fusco S , Abermann E , Fink C . The posterior cruciate ligament angle in the setting of anterior cruciate ligament deficient knees: the effect of gender, age, time from injury and tibial slope. Radiol Med. 2025;130:534–542. 39863738 10.1007/s11547-025-01951-x PMC12008072

[8] Fornalski S , McGarry MH , Csintalan RP , Fithian DC , Lee TQ . Biomechanical and anatomical assessment after knee hyperextension injury. Am J Sports Med. 2008;36:80–84. 17932409 10.1177/0363546507308189

[9] Fowler PJ , Messieh SS . Isolated posterior cruciate ligament injuries in athletes. Am J Sports Med. 1987;15:553–557. 3425783 10.1177/036354658701500606

[10] Fritz B . Bildgebung des vorderen Kreuzbands und der anterolateralen Rotationsinstabilität desKniegelenks. Radiologie. 2024;64:261–270. 38441595 10.1007/s00117-024-01278-0 PMC10973086

[11] Geeslin AG , LaPrade RF . Location of bone bruises and other osseous injuries associated with acute grade III isolated and combined posterolateral knee injuries. Am J Sports Med. 2010;38:2502–2508. 20837553 10.1177/0363546510376232

[12] Grover JS , Bassett LW , Gross ML , Seeger LL , Finerman GA . Posterior cruciate ligament: MR imaging. Radiology. 1990;174:527–530. 2296661 10.1148/radiology.174.2.2296661

[13] Halewood C , Amis AA . Clinically relevant biomechanics of the knee capsule and ligaments. Knee Surg Sports Traumatol Arthrosc. 2015;23:2789–2796. 25894747 10.1007/s00167-015-3594-8

[14] Hassebrock JD , Gulbrandsen MT , Asprey WL , Makovicka JL , Chhabra A . Knee ligament anatomy and biomechanics. Sports Med Arthrosc. 2020;28:80–86. 32740458 10.1097/JSA.0000000000000279

[15] Hochstein P , Schmickal T , Grützner PA , Wentzensen A . Diagnostic and incidence of the rupture of the posterior cruciate ligament. Unfallchirurg. 1999;102:753–762. 10525618 10.1007/s001130050477

[16] Ibanez M , Valcarenghi J , Hoffmann F , Mouton C , Pioger C , Siboni R , et al. Nonanatomic posteromedial bundle augmentation of the posterior cruciate ligament after hyperextension trauma. Arthrosc Tech. 2024;13:103013. 39233795 10.1016/j.eats.2024.103013 PMC11369956

[17] Kopkow C , Freiberg A , Kirschner S , Seidler A , Schmitt J . Physical examination tests for the diagnosis of posterior cruciate ligament rupture: a systematic review. J Orthop Sports Phys Ther. 2013;43:804–813. 24175598 10.2519/jospt.2013.4906

[18] LaPrade CM , Civitarese DM , Rasmussen MT , LaPrade RF . Emerging updates on the posterior cruciate ligament: a review of the current literature. Am J Sports Med. 2015;43:3077–3092. 25776184 10.1177/0363546515572770

[19] LaPrade RF , Floyd ER , Falaas KL , Ebert NJ , Struyk GD , Carlson GB , et al. The posterior cruciate ligament: anatomy, biomechanics, and double‐bundle reconstruction. J Arthrosc Surg Sports Med. 2021;2:94–107.

[20] Lee BK , Nam SW . Rupture of posterior cruciate ligament: diagnosis and treatment principles. Knee Surg Relat Res. 2011;23:135–141. 22570824 10.5792/ksrr.2011.23.3.135 PMC3341837

[21] Lundblad M , Hagglund M , Thomee C , Hamrin Senorski E , Ekstrand J , Karlsson J , et al. Epidemiological data on LCL and PCL injuries over 17 seasons in men's professional soccer: the UEFA Elite Club Injury Study. Open Access J Sports Med. 2020;11:105–112. 32494208 10.2147/OAJSM.S237997 PMC7231769

[22] Mouton C , Ibañez M , Hoffmann F , Monllau JC , Seil R . Injuries of the posteromedial bundle of the posterior cruciate ligament after knee hyperextension trauma: a new clinical entity based on an original case series. J Exp Orthop. 2024;11:e12052. 38974050 10.1002/jeo2.12052 PMC11224766

[23] Naraghi AM , White LM . Imaging of athletic injuries of knee ligaments and menisci: sports imaging series. Radiology. 2016;281:23–40. 27643766 10.1148/radiol.2016152320

[24] Oronowicz J , Mouton C , Pioger C , Valcarenghi J , Tischer T , Seil R . The posterior cruciate ligament‐posterior femoral cortex angle (PCL‐PCA) and the lateral collateral ligament (LCL) sign are useful parameters to indicate the progression of knee decompensation over time after an ACL injury. Knee Surg Sports Traumatol Arthrosc. 2023;31:5128–5136. 37805550 10.1007/s00167-023-07583-w

[25] Park HJ , Lee SY , Choi YJ , Choi SH , Kim MS , Ahn JH , et al. The usefulness of the oblique coronal plane of three‐dimensional isotropic T2‐weighted fast spin‐echo (VISTA) knee MRI in the evaluation of posterior cruciate ligament reconstruction with allograft: comparison with the oblique coronal plane of two‐dimensional fast spin‐echo T2‐weighted sequences. Eur J Radiol. 2019;114:105–110. 31005159 10.1016/j.ejrad.2019.03.009

[26] Parkar AP . Imaging the anterior and posterior cruciate ligaments. J Bel Soc Radiol. 2016;100:98. 10.5334/jbr-btr.1197 PMC6100662 30151491

[27] Parkar AP , Adriaensen MEAPM . ESR essentials: MRI of the knee‐practice recommendations by ESSR. Eur Radiol. 2024;34:6590–6599. 38536461 10.1007/s00330-024-10706-7 PMC11399221

[28] Paschos NK . Editorial commentary: the posterior cruciate ligament posteromedial bundle is small but vital to posterior cruciate ligament biomechanics: don't ignore the underdog. Arthroscopy. 2020;36:2885–2887. 33172585 10.1016/j.arthro.2020.08.019

[29] Rodriguez Jr, W , Vinson EN , Helms CA , Toth AP . MRI appearance of posterior cruciate ligament tears. Am J Roentgenol. 2008;191:W155–W159. 10.2214/AJR.07.2921 18806138

[30] Schroven W , Vles G , Verhaegen J , Roussot M , Bellemans J , Konan S . Operative management of isolated posterior cruciate ligament injuries improves stability and reduces the incidence of secondary osteoarthritis: a systematic review. Knee Surg Sports Traumatol Arthrosc. 2022;30:1733–1743. 34505176 10.1007/s00167-021-06723-4

[31] Siboni R , Pioger C , Mouton C , Seil R . The posterior cruciate ligament‐posterior femoral cortex angle: a reliable and accurate MRI method to quantify the buckling phenomenon of the PCL in ACL‐deficient knees. Knee Surg Sports Traumatol Arthrosc. 2023;31:332–339. 36057669 10.1007/s00167-022-07145-6

[32] Vohra S , Arnold G , Doshi S , Marcantonio D . Normal MR imaging anatomy of the knee. Magn Reson Imaging Clin N Am. 2011;19:637–653. 21816336 10.1016/j.mric.2011.05.012

[33] Wang SH , Chien WC , Chung CH , Wang YC , Lin LC , Pan RY . Long‐term results of posterior cruciate ligament tear with or without reconstruction: a nationwide, population‐based cohort study. PLoS One. 2018;13:e0205118. 30281658 10.1371/journal.pone.0205118 PMC6169976

[34] Winkler PW , Zsidai B , Wagala NN , Hughes JD , Horvath A , Senorski EH , et al. Evolving evidence in the treatment of primary and recurrent posterior cruciate ligament injuries, part 1: anatomy, biomechanics and diagnostics. Knee Surg Sports Traumatol Arthrosc. 2021;29:672–681. 33201271 10.1007/s00167-020-06357-y PMC7917041

[35] Winkler PW , Zsidai B , Wagala NN , Hughes JD , Horvath A , Senorski EH , et al. Evolving evidence in the treatment of primary and recurrent posterior cruciate ligament injuries, part 2: surgical techniques, outcomes and rehabilitation. Knee Surg Sports Traumatol Arthrosc. 2021;29:682–693. 33125531 10.1007/s00167-020-06337-2 PMC7917042

[36] Winters K , Tregonning R . Reliability of magnetic resonance imaging of the traumatic knee as determined by arthroscopy. N Z Med J. 2005;118(U):1301. 15711634

[37] Zsidai B , Horvath A , Winkler PW , Narup E , Kaarre J , Svantesson E , et al. Different injury patterns exist among patients undergoing operative treatment of isolated PCL, combined PCL/ACL, and isolated ACL injuries: a study from the Swedish National Knee Ligament Registry. Knee Surg Sports Traumatol Arthrosc. 2022;30:3451–3460. 35357530 10.1007/s00167-022-06948-x PMC9464165

